# DO PROXIMAL AND DISTAL GASTRIC TUMOURS BEHAVE DIFFERENTLY?

**DOI:** 10.1590/0102-6720201600040005

**Published:** 2016

**Authors:** Laurence Bedin da COSTA, Marcelo Garcia TONETO, Luis Fernando MOREIRA

**Affiliations:** 1 Postgraduate Program in Medicine, Surgical Sciences, Universidade Federal do Rio Grande do Sul; RS, Brazil; 2 Department of Surgery, Hospital São Lucas, Pontifícia Universidade Católica do Rio Grande do Sul; RS, Brazil; 3 Department of Surgery, Hospital de Clínicas de Porto Alegre; Porto Alegre, RS, Brazil.

**Keywords:** Stomach neoplasms, Prognosis, Survivorship, Mortality, Lymph Node Excision.

## Abstract

**Background:**

Although the incidence of gastric (adenocarcinoma) cancer has been decreasing over time, it is still one of the most common malignancies worldwide, and proximal tumours tend to have a worse prognosis.

**Aim:**

To compare surgical outcomes and prognosis between proximal - excluding tumours of the cardia - and distal gastric cancer.

**Methods:**

Out of 293 cases reviewed - 209 with distal and 69 with proximal gastric cancer - were compared for clinical and pathological features, stage, surgical outcome, mortality and survival.

**Results:**

Statistically, there was no significant difference between patients in both groups regarding mortality (p=0.661), adjuvant chemotherapy (p 0.661), and radiation (p=1.000). However, there was significant difference in the degree of lymph node dissection employed (p=0.002) and the number of positive lymph nodes resected (p=0.038) between the two groups. The odds of death at five years for patients who had a D0 dissection was three times greater (odds ratio 2.78; (95%CI 1.33-5.82) than that for patients who had a D2 dissection, while for patients who had a D1 dissection the odds ratio was only 1.41 (95%CI 0.71-2.83) compared to D2-dissected patients.

**Conclusion:**

Although no significant differences were found between proximal and distal gastric cancer, the increased risk of death in D0- and D1-dissected patients clearly suggests an important role of radical D2 lymph node dissection in survival.

## RESUMO

**Racional:**

Embora a incidência do câncer gástrico esteja diminuindo nas últimas décadas, ele ainda aparece como uma das neoplasias malignas mais comuns, e tumores proximais tendem a ter pior prognóstico.

**Objetivo:**

Comparar os resultados cirúrgicos e o prognóstico entre o câncer gástrico proximal, excluindo os tumores da cárdia e junção esofagogástrica, e o distal.

**Métodos:**

De 293 casos revistos - 209 distais e 69 proximais - foram comparados quanto aos achados clínicos e patológicos, estágio, resultados cirúrgicos, mortalidade e sobrevida.

**Resultados:**

Estatisticamente não houve diferença entre pacientes em ambos os grupos quanto à mortalidade (p=0.661), emprego de quimioterapia adjuvante (p=0.661) e de radioterapia (p=1.000). Entretanto, houve diferença significativa no grau de dissecção linfonodal empregada (p=0.002) e no número de linfonodos positivos ressecados (p=0.038) entre os dois grupos. A razão de chances para morte em cinco anos nos casos de dissecção D0 foi três vezes maior (2,78; IC95% de 1,33 a 5,82) do que a D2, enquanto que para dissecção D1, ela foi apenas 1,41 vezes maior (95%CI 0.71-2.83) quando comparado à D2.

**Conclusão:**

Ainda que não se tenha observado diferenças significativas entre o câncer gástrico proximal e o distal, o risco de morte aumentado nos casos de D0 e D1, claramente demonstra o papel preponderante da linfadenectomia radical D2 no tratamento dessa doença.

## INTRODUCTION

Although the incidence of gastric cancer has decreased over the past decades, it still remains a relevant problem, being among the most common malignancies worldwide. According to the Globocan^8^ 2014 project from the World Health Organization (WHO), there were approximately a million new gastric cancer cases worldwide (952,000 cases; 7% of all malignancies), ranking gastric cancer as the 5^th^ most common tumor in absolute numbers. In Brazil, data from the National Cancer Institute for 2016 place gastric cancer as the 4^th^ most common cancer in men (12,870 cases) and the 6^th^ most common in women (7,520 cases).These figures place the stomach as the 6^th^ most frequently organ affected by cancer in Brazil^10^.

In the past, tumors originating in the cardia and in the gastro-esophageal junction were usually addressed as proximal gastric tumors, indistinctly. The anatomical structure of the proximal third of the stomach, where the serosa is partially developed, increasing the likelihood that these tumors will be diagnosed at a more advanced stage, may also be associated with unfavorable prognosis on proximal tumors^1^.

Moreover, there is no clear agreement on the link between mortality and tumor location in the stomach. Earlier papers considered prognostic and survival differences and stated that cancers originating in the cardia and in the gastro-esophageal junction tended to have a worse prognosis than those affecting more distal portions of the organ^13^ ^,^ ^27^. 

However, some authors have shown that, when cases are analyzed at sub-stages, outcomes are similar^14^ ^,^ ^23^ ^,^ ^24^. Still, excluding the tumors located either in the gastro-esophageal junction with esophageal predominance or those affecting primarily the anatomical cardia (Siewert I and II types), no significant differences were observed on survival among primary tumors originated at the upper, middle, or lower stomach^25^ ^,^ ^26^.

Nowadays, when there is a tendency to proximal migration of the primary tumor in the stomach, those parameters and differences between proximal and distal tumor need to be revised.

This study was designed to analyze both surgical and oncologic findings and outcomes of gastric cancer, and to compare differences between proximal (excluding tumors from esophagogastric origin) vs. distal lesions.

## METHODS

This was a retrospective cohort study of 293 patients with adenocarcinoma of the stomach who underwent treatment at a university hospital (São Lucas Hospital in the Pontifícia Universidade Católica), located in the city of Porto Alegre, RS, Brazil, from January 2002 to January 2015. Patients' medical records from the Medical File Service of the institution were used as a research source. Cases with missing or incomplete data as well as those with histopathological findings other than adenocarcinoma and Siewert tumors types I and II were excluded from the analysis.

Preoperative endoscopy, pathology and surgical reports were reviewed, and tumor location was classified according to the criteria of the Japanese Gastric Cancer Association^11^. Proximal gastric cancer (PGC) was considered when the tumor extended from one point to more than 2 cm distal to the gastro-esophageal junction (Siewert type III) up to a crossing line between the left gastric artery and the end of the left gastroepiploic artery. Tumors below this crossing line were considered distal tumors. Demographic and epidemiological data, such as age, gender, tumor size, and number of dissected and involved lymph nodes were collected. 

Tumor staging followed the guidelines of the TNM (tumor-node-metastasis) system of the American Joint Committee on Cancer (AJCC)^7^, 7^th^ edition.

Postoperative surgical complications, excluding those that occurred after discharge, were classified according to the system proposed by Clavien, in 1992, and modified by Dindo, in 2004^4^.

### Statistical analysis

Quantitative data were expressed as mean and standard deviation, or median and minimum-maximum ranges, according to variable distribution. As for qualitative data, absolute frequencies and percentage were used. Distal and proximal cases were analyzed using the Wilcoxon-Mann-Whitney test and the Chi-Square test, followed by analysis of residues, if needed. Cox Regression was used to compare survival between patients with proximal and distal tumors, respectively. The Kaplan-Meier method was used to estimate survival as a function of time, and the Log-Rank test was used for comparison of survival curves according to clinic-pathological characteristics. Statistical analysis was performed with the help of the Statistical Package for the Social Sciences (SPSS), version 18.0^22^, and the level of significance was set at p<0.05.

## RESULTS

A total of 293 patients diagnosed with adenocarcinoma of the stomach were initially included in the study. A hundred and eighty-eight (64%) were men and 105 (36%) women. In 15 cases, initial tumor location could not be determined accurately enough to be included in the study. Five of them classified as multifocal, four as anastomotic recurrence, and in six cases, available data were not reliable. Therefore, 278 patients, 69 (25%) of them with PGC and 209 (75%) with distal gastric cancer (DGC) were included. Table 1 shows the clinic-pathological findings of the patients.

**TABLE 1:**
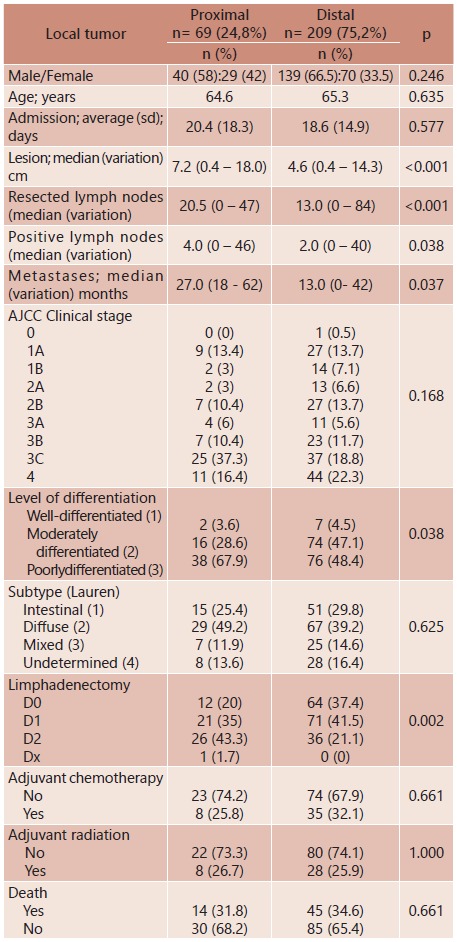
Clinic-pathological findings of patients depending on the location of primary tumor in the stomach

In the PGC group, the median range number of lymph nodes removed was 20 (0-47), significantly lower (p<0.001 than in the DGC group, which was 13 (0-84). Positive range lymph nodes were also lower in the PGC group as compared to the DGC group, 2 (0-40), and 4 (0-46), respectively (p=0.038). As for lymph node status, the probability of being alive in five years was 48% (11%) for N1 (n=34), 37% (11%) for N2 (n=41), and 23% (7%) for N3 (n=73), with no significant difference between these groups, statistically.

Patients who underwent D0 lymph node dissection had a median survival of 26 months, which was less than that of patients who underwent D1 dissection (54 months) or D2 dissection (63 months). Moreover, for patients with a D0 lymph node dissection, the cumulative probability of being alive in five years was 30% (8%), while for those who had a D1 or D2 dissection, these probabilities were 48% (7%) and 53% (9%), respectively. However, differences in survival between the levels of lymph node dissection were not statistically significant.

**FIGURE 1:**
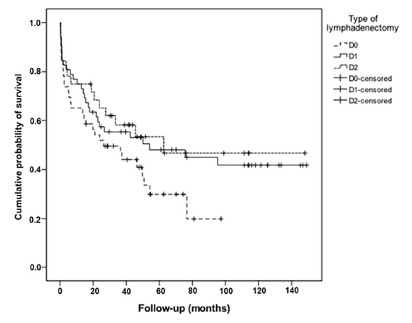
Survival according to the type of lymphadenectomy

Figure 1 shows the overall survival curve according to the level of lymph node dissection performed.

As expected, the majority of patients in both groups had advanced tumors. Proximal tumors were larger in size (p<0.001), presented with a higher number of positive lymph nodes (p=0.038), and were significantly less differentiated, as for histopathology, than distal lesions (p=0.038). Early lesions (T1) accounted for 19% of all tumors (16% of PGC and 20% of DGC). On the other hand, 20% of patients had disseminated tumor already (M1) at diagnosis, also 16% and20%, respectively.

Overall, average (SD) tumor size was 5.8 (3.7 cm), ranging from 4 mm to 20 cm. Average (SD) size in the DGC and PGC groups were 7.4 (4.3 cm) and 5 (2.8 cm), respectively (p<0.001).Tumor size was directly related to mortality, where patients with tumors larger than 8 cm in diameter had a significantly shorter survival than those with lesions smaller than 5 cm (6 vs. 50 months; p<0.001). Furthermore, patients with tumors larger than 8 cm died earlier within 60 months (81% vs. 53%) as compared to those patients with lesions smaller than 5 cm. The probability of being alive in five years was 14% (4.7%) for patients with tumors larger than 8 cm, while for patients with tumors smaller than 5 cm, this probability was 42% (7%).Statistically, the effect of tumor size on survival did not differ between proximal and distal locations.

As for factors related to survival, T4 tumors were found to be strongly positively associated with N3 lymph node involvement (p=0.001), where more than half (58%) of T4 cases were N3. The majority of T2/T3 tumors were associated with a lower lymph node involvement (N1 and N2), and only a small percentage of them (8%) were N3.

Patients with T4 tumors (n=158) had the worst outcomes, and the probability of being alive in five years was 32% (5.4%). When cases with tumors confined into the muscle or up to the serosa (T2/T3) were analyzed together (n=47), the probability of being alive in five years was 47% (11.4%).T4 cases had a median survival of only 21 months, while T2/T3 cases had a median survival of 54 months, but this difference was not statistically significant. Patients with T4 tumors presented 2.17 times [0.94-5.02; 95% CI] more chances to die within five years than patients with lesions with less impairment of the organ wall.

There were postoperative complications in 25% of patients with PGC and 23% of patients with DGC. The most common complication in both groups was pneumonia, which affected 30 (14%) patients, followed by fistula in 17 (8%), sepsis in 8 (4%), and wound infection in 6 (2.8%). Intra hospital mortality rate was 5% (n=6).

Overall recurrence was 37%; 48 out of 128 cases were followed up till the end of the study, being peritoneal surface (n=29) and liver (n=11) the most common sites. There was no statistically significant difference between the two groups regarding the use of adjuvant therapy, where 11 (8%) patients receiving chemotherapy alone and 31 (23%) receiving also radiotherapy did relapse.

Survival rates were calculated based on the follow-up of 278 patients, and cumulative probability of being alive in five years was estimated in 35% (7.4%) for patients with PGC and 32% (4.5%) for patients with DGC. This difference was not statistically significant between both groups either.

**FIGURE 2:**
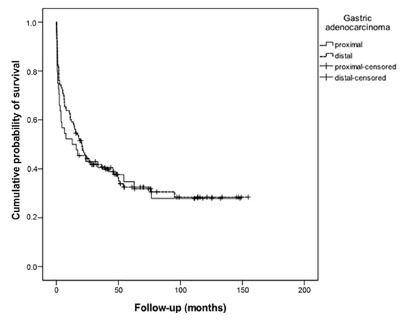
Survival according to the location of primary tumor

Figure 2 shows the overall survival curve for patients in the two groups throughout study period. When adjusted for tumor size and degree of lymph node dissection performed, the higher the number of positive lymph nodes, the greater the mortality (p=0.007).

On multivariate analysis of survival at 60 months, considering tumor location (proximal vs. distal), T4 category (T4 vs. T2/T3), N category (N0 vs. N1 vs. N2), and the degree of lymphadenectomy (D0 vs. D1 vs. D2) as related factors, the only significant risk factor, following adjustments, was the degree of lymphadenectomy performed (p=0.018). 

Patients who had a D0 lymphadenectomy presented 2.8 [95% CI; 1.33-5.82] times higher death risk in five years than D2-dissected patients, while patients who had a D1 lymphadenectomy presented 1.4 [95% CI; 0.71-2.83] times higher death risk in five years than the D2 death cases by the end of the same period of time.

Overall mortality was 41% (n=115), where 30 (43%) and 85 (41%) patients died in the PGC and DGC groups, respectively. Out of 69 patients with PGC and 209 patients with DGC, 39 (57%) and 124 (59%) were still alive at the end of the study period. There was no significant difference in mortality between the two groups.

Besides that, there was no difference in mortality based on location of primary lesion, as cumulative probability of being alive in five years was 35% (14.5%) for the PGC group, and 32% (9%) for the DGC group.

## DISCUSSION

It is well known that aggressiveness of gastric cancer depends on a number of factors, including wall invasion pattern and lymph node status, which are the two most important findings to estimate prognosis and to guide decision making^17^ ^,^ ^19^. Furthermore, the presence of regional lymph node metastases alone is the most important independent prognostic factor for survival in these patients^3^ ^,^ ^6^. Although proximal lesions did show deeper invasion and higher lymph node involvement, outcomes did not differ significantly between the two groups.

Does tumor size matter? In patients with other types of cancer, such as breast or lung cancer, tumor size is still an important predictor. In gastric cancer, however, the prognostic value of tumor size remains controversial^16^ ^,^ ^29^. Some authors showed that tumor size can be an important factor as for aggressiveness^9^ ^,^ ^30^. This is consistent with the present study, where a direct relationship was found between lesion diameter and aggressiveness. The larger the tumor, the more aggressive, with deeper invasion in stomach wall and higher degree of lymph node involvement. Nevertheless, proximal lesions were significantly larger than distal ones.

Treatment of gastric cancer is multi-factorial, but surgery still plays a primary role in management, since it is the only approach that can lead to cure. The stage at which the disease is detected plays a crucial role in treatment choice. The use of chemotherapy or radiotherapy combined with surgery is well established, with clear benefits in survival and disease progression. This approach was strengthened especially after the classic study by Macdonald et al. in 2001^18^, with the combination of preoperative chemotherapy and postoperative radiation, which showed an increase in overall survival from 27 to 36 months, compared to surgery alone. Cunningham, in 2006^5^, showed improvement in overall and progression-free survival with per-operative chemotherapy.

Nevertheless, prognosis of gastric cancer is still quite dismal - despite constant improvements in adjuvant therapy with the development of new drugs and association of target therapy that have, undoubtedly, improved survival -, especially for those cases with serosal invasion. In such cases, even after radical resection, about 20-40% of the patients die due to recurrence^2^ ^,^ ^20^ and present peritoneal dissemination, which is, in fact, the main cause of treatment failure, and where serosal invasion is a predisposing factor^15^. In this study, no difference was found on peritoneal recurrence according to primary tumor location, and this was not affected by either preoperative or postoperative adjuvant treatment.

Overall survival in five years for patients operated on for advanced gastric cancer differs among countries and medical institutions, but it is generally below 30% in Western countries, such as those of Europe and in the United States^11^ ^,^ ^28^. In Asian countries, such as Japan and South Korea, survival is significantly higher, reaching approximately 70%^21^. In the present study, survival was identical in both groups, with a rate of 37% for PGC and 36% for DGC, consistent with published results.

Despite therapeutic advances over the past decades, especially regarding adjuvant therapy, mortality of gastric adenocarcinoma remains high. The results of the present study showed no significant differences in surgical outcome or prognosis between proximal and distal lesions. As expected, and similar to previous studies, the degree of lymph node involvement and tumor size were independent factors that affected survival. 

## CONCLUSION

Although significant differences between distal and proximal gastric tumors have not been observed, the increased risk of death in D0 and D1-dissected cases clearly suggests a main role of radical D2 lymphadenectomy in the treatment of this disease.
